# Limited effect of surfactant protein D ablation on doxorubicin-induced intestinal toxicity in mice

**DOI:** 10.3389/fimmu.2026.1913868

**Published:** 2026-09-10

**Authors:** Mathias Rathe, Sebastian von Huth, Faisal Fazli, Mark Burton, Mads Thomassen, Pingping Jiang, Jesper B. Moeller, Uffe Holmskov, Steffen Husby, Grith L. Sorensen

**Affiliations:** 1 Hans Christian Andersen Children’s Hospital, Odense University Hospital, Odense, Denmark; 2 Department of Clinical Research, University of Southern Denmark, Odense, Denmark; 3 Department of Molecular Medicine, University of Southern Denmark, Odense, Denmark; 4 Department of Clinical Genetics, Odense University Hospital, Odense, Denmark; 5 Section of Comparative Pediatrics and Nutrition, Department of Clinical Veterinary and Animal Science, University of Copenhagen, Copenhagen, Denmark; 6 Danish Institute for Advanced Study , University of Southern Denmark, Odense, Denmark

**Keywords:** chemotherapy, doxorubicin, gastrointestinal toxicity, mucositis, murine, SP-D, surfactant protein D

## Abstract

**Introduction:**

Surfactant protein (SP-D) is an anti-inflammatory host defense molecule, produced by epithelial cells and present on mucosal surfaces throughout the human and mouse body. In the present study, we investigated whether SP-D attenuates chemotherapy-induced gastrointestinal toxicity and inflammation in mice.

**Methods:**

SP-D knockout (KO, *Sftpd*^-/-^) mice and wild type (WT, *Sftpd*^+/+^) littermates were treated with doxorubicin (20mg/kg) or saline and sacrificed at day 3 o rat day 7, post-administration. Gastrointestinal toxicity was evaluated by weight change, citrulline levels, and histopathology. Genetic expression of genes related to chemotherapy-induced mucositis, inflammation, apoptosis, and repair of the damaged epithelium was investigated by reverse transcription quantitative polymerase chain reaction (RT-qPCR) on jejunal and colonic samples.

**Results:**

Doxorubicin significantly reduced bodyweight, and KO mice lost significantly more weight relative to WT mice. Transcriptional analyses showed a significant SP-D-dependent increase of gasdermin genes (*Gsdmc2/3* and *Gsdmc4*) in the small intestine. The doxorubicin challenge did not alter intestinal SP-D expression.

**Discussion:**

In conclusion, SP-D had a limited but significant impact on mouse gastrointestinal toxicity after chemotherapy-induced mucositis, which primarily involved weight loss of unknown cause and had no or limited effects on intestinal morphology and inflammation.

## Introduction

Surfactant protein D (SP-D) is a host defense molecule produced by the innate immune system that enhances pathogen clearance and modulates inflammatory responses, mucosal immunity, and host defense against infections (1, 2). SP-D is produced by epithelial cells, and although SP-D is primarily known for its role in pulmonary innate immunity (2), SP-D is also widely distributed in extrapulmonary mucosal surfaces, suggesting that this protein plays an important role in the first line of host defense against invading pathogens and in directing inflammatory responses in various tissues (3–5). In this regard, we have previously shown a strong positive correlation between inflammatory activity and epithelial expression of SP-D in surgical specimens from inflammatory bowel disease (IBD) patients, supporting a modulatory role of SP-D in intestinal inflammation (6). Moreover, genetic variation of SP-D has been associated with IBD (7, 8). Specific roles of intestinal SP-D have been demonstrated to include the preservation of tight junction protein structures under inflammatory conditions and protection against dysbiosis (9, 10). Moreover, SP-D is thought to regulate NF-κB–induced inflammation, matrix metalloproteinase (MMP) production, and lipopolysaccharide (LPS)-induced TLR4–mediated inflammation by inhibiting LPS-induced inflammatory responses and by scavenging pathogenic intestinal bacteria (11–14).

We previously observed that SP-D expression was upregulated in the intestinal mucosa of piglets treated with doxorubicin (15). The upregulation of SP-D may have implications for chemotherapy-induced mucositis, a common, debilitating adverse effect occurring during cancer treatment with severe implications for the morbidity and mortality of cancer patients (16, 17).

Chemotherapy-induced intestinal mucositis is a complex process involving direct and indirect effects of cytotoxic treatment on the gastrointestinal mucosa. Chemotherapeutic agents preferentially affect rapidly proliferating intestinal epithelial cells, resulting in epithelial apoptosis, impaired mucosal integrity, and disruption of the intestinal barrier. These initial events can subsequently trigger activation of innate immune and inflammatory pathways, leading to the release of pro-inflammatory mediators, recruitment and activation of immune cells, and further amplification of tissue injury. The resulting gastrointestinal dysfunction can manifest as reduced nutrient absorption, diarrhea, abdominal pain, and weight loss, and may lead to dose reductions or treatment interruptions that compromise the delivery of effective cancer therapy (16, 17). The intestinal mucosa therefore represents an important determinant of the overall tolerability and clinical outcome of chemotherapy, and identifying endogenous mechanisms that promote mucosal protection and recovery may provide new opportunities for reducing chemotherapy-associated gastrointestinal toxicity.

The pathophysiological mechanisms underlying chemotherapy-induced mucositis involve several processes in which SP-D may potentially participate. As a component of mucosal innate immunity, SP-D may influence epithelial integrity, inflammatory signaling, and host-microbiota interactions. In this way, SP-D could potentially attenuate the inflammatory amplification of chemotherapy-induced mucosal damage. However, whether endogenous SP-D has a functional role in the intestinal response to cytotoxic chemotherapy remains unclear.

In summary, SP-D interacts with pathways potentially involved in inducing and perpetuating mucositis. We hypothesized that SP-D might attenuate doxorubicin chemotherapy-induced intestinal inflammation, thereby reducing gastrointestinal complications after chemotherapeutic treatment, and aimed to investigate this using a doxorubicin-challenged mouse model of gastrointestinal mucositis.

## Materials and methods

### Animals

We used adult 10-12-week-old female *Sftpd^-/-^* (knockout/KO) mice backcrossed more than 10 times onto the C57BL/6N strain, and *Sftpd^+/+^* (wild-type/WT) littermates. All mice were genotyped and bred in-house. The KO mice have previously been shown to be deficient in SP-D mRNA and protein expression (18). The genotypes of KO and WT mice were confirmed by DNA PCR.

To familiarize the animals with individual housing and daily handling, mice were housed in filter-top cages containing aspen woodchips bedding, nesting material, tunnel houses, and aspen chew to provide environmental enrichment one week before mucositis induction. Mice were housed under diurnal lighting conditions with free access to regular chow and water.

### Murine model of doxorubicin-induced intestinal mucositis

A total of 69 mice were included to investigate the effect of SP-D on doxorubicin-induced gastrointestinal toxicity. At day 0, SP-D KO mice and their WT littermates were randomized to receive either a single intraperitoneal injection of doxorubicin 20 mg/kg (Doxorubicin Accord, Accord Healthcare Limited, North Harrow, Middlesex, England) or an equal volume of isotonic saline. This dose has previously been shown to result in tolerable body weight loss and a mild reduction in small intestinal length in mice, and therefore represents a relevant experimental window (19). Mice were further assigned for sacrifice on either day 3 or day 7.

Three mice (1 WT and 2 KO) receiving doxorubicin were terminated prior to endpoint due to excessive weight loss. Eight experimental groups of littermates were investigated:

WT receiving saline and terminated at day 3 (WT-NaCl3), n = 5
WT receiving saline and terminated at day 7 (WT-NaCl7), n = 5
WT receiving doxorubicin and terminated at day 3 (WT-Dox3), n = 10
WT receiving doxorubicin and terminated at day 7 (WT-Dox7), n = 12
KO receiving saline and terminated at day 3 (KO-NaCl3), n = 6
KO receiving saline and terminated at day 7 (KO-NaCl7), n = 6
KO receiving doxorubicin and terminated at day 3 (KO-Dox3), n = 10
KO receiving doxorubicin and terminated at day 7 (KO-Dox7), n = 12

Mice were anesthetized with an intraperitoneal injection of ketamine 100 mg/kg (Ketaminol^®^ Wet, MSD Animal Health, Copenhagen, Denmark) and xylazine 10 mg/kg (Rompun^®^ Vet, Bayer A/S, AnimalHealth, Copenhagen, Denmark). The animals were euthanized by cervical dislocation. The small intestine (from the pyloric sphincter to the ileocecal junction) was removed, measured, and divided into three segments of equal length, designated proximal, middle, and distal regions, respectively. The colon was removed, its length was recorded, and tissue samples were obtained. For each intestinal region, a 0.5 cm section was fixed in 4% neutral buffered formalin for 48 hrs. After fixation, the tissue was embedded in paraffin and further cross-sectioned (5 µm), mounted on glass slides, and stained with hematoxylin and eosin (HE). Additional tissue samples from each intestinal region were snap-frozen in liquid nitrogen for subsequent analyses. Finally, a piece of sternum containing bone marrow was collected for evaluation of cellularity. The sternum was decalcified in buffered formic acid (Kristenson’s solution) prior to HE-staining.

### RNA purification

Total RNA was isolated using TRIzol (Invitrogen Life Technologies, Carlsbad, CA, USA) according to the manufacturer’s instructions. RNA was quantified, and the purity was confirmed by spectrophotometry using the NanoDrop (Thermo Scientific, Wilmington, DE, USA). All samples had A260/280 nm ratios ≥ 1.8, suggesting adequate RNA purity. For samples used in the micro array experiment integrity was verified using the Agilent 2100 Bioanalyzer and the Agilent RNA 6000 nano kit (Santa Clara, CA, USA).

### Microarray analysis

RNA samples from jejuna (n = 5/condition (total n = 40)) were analyzed using Affymetrix GeneChip^®^ Mouse Transcriptome Array 1.0. Hybridization, washing, staining, and scanning were performed automatically in an Affymetrix GeneTitan instrument. Array data preprocessing was performed using the R statistical package *limma*. Raw intensity data were background corrected using the *normexp* method. Data normalization was done by the *quantile* method. Analysis of variance (ANOVA) was used for the identification of significantly differentially regulated genes between conditions using the Transcriptome Analysis Console v3.0. To correct for multiple comparisons, only genes with a false discovery rate (FDR)-corrected P-value of < 0.05 were considered statistically significant. Pathway analysis was performed by parametric analysis of gene set enrichment using the *PGSEA* R-package. To validate the general gene signature, we searched the Gene Expression Omnibus for relevant datasets containing microarray gene expression data from the jejunum following chemotherapy exposure. We identified one relevant dataset (GEO dataset – GSE11722) providing array data from the GI tracts of 6- to 8-week-old rats up to 72 h after treatment with the chemotherapeutic agent irinotecan (20). This allowed exploration of similarities in temporal and region-specific changes in gene expression by using gene set enrichment analysis (GSEA) as described by Subramanian et al. (21).

### Quantitative real-time PCR of genes related to mucositis, inflammation, and damaged epithelium

We evaluated genes related to chemotherapy-induced mucositis, NF-κB-mediated inflammation, and apoptosis, as well as genes indigenous to the gastrointestinal tract and/or involved in wound healing or repair of damaged epithelium (22–24). The evaluation of genes was an RT-qPCR–based analysis including 11 genes related to mucositis, inflammation, tissue injury/repair and apoptosis. We investigated the expression of *Tnf*, *Il-1β*, *Casp-1*, *Casp-3*, and Bcl-2 associated X protein (*Bax*). We investigated *Mmp2* and *Mmp12* both associated with SP-D and chemotherapy-induced mucositis (12, 25). We further investigated the effect on five genes indigenous to the gastrointestinal tract and/or involved in wound healing and repair of damaged epithelium (*Serpina3n*, *Akr1b8*, *Gsdmc2*, *Gsdmc3*, *Gsdmc4*) (22–24).

Gene candidates for validation were selected based on microarray gene expression profiles (**Supplementary Table 1**), including significantly regulated genes related to NF-κB-mediated inflammation. Furthermore, we selected five of the top ten most strongly regulated genes (up- or downregulated), indigenous to the gastrointestinal tract and/or involved in epithelial repair. All genes were investigated at day 3 in both the jejunum and colon. Moreover, SP-D (*Sftpd*) gene expression was assessed in colon samples. Assay identification numbers and reference gene numbers of the target and housekeeping gene are shown in **Supplementary Table 2**.

Complementary DNA (cDNA) synthesis was performed using the M-MLV Reverse Transcriptase kit (Sigma-Aldrich, Taufkirchen, Germany), according to manufacturer’s instructions. Synthesized cDNA was used as a template for real-time PCR. TaqMan^®^ Universal Mastermix II and Gene Expression Assays were used (Applied Biosystems™, Foster City, CA, USA). Reactions were performed in technical triplicates, using a StepOnePlus real-time PCR system (Life Technologies, Carlsbad, CA, USA). Relative mRNA levels were calculated using the ΔΔCt-method. The relative expression was analyzed using the qBase software (Biogazelle, Zwijnaarde, Belgium) using the housekeeping gene *Gapdh*.

### RT-qPCR of *Sftpd* expression in jejunum, colon, intestinal epithelial cells (IECs), and lamina propria lymphocytes

Intestinal tissues for quantification of *Sftpd* mRNA expression were obtained from 8-10-week-old male C57BL/6J mice (The Jackson Laboratory). Isolation of intestinal epithelial cells (IECs) and lamina propria leukocytes (LPLs) was performed as previously described (26). cDNA synthesis was performed using random hexamers and the Superscript III reverse transcriptase according to the manufacturer’s recommendations (ThermoFisher). Quantification of gene expression by real-time PCR was performed on a QuantStudio 6 Flex Real-time PCR system (ThermoFisher). Quantification of SP-D gene expression was achieved using TaqMan assay Mm00486060 (ThermoFisher). The relative expression was analyzed using the housekeeping genes *Gapdh* and *Tbp*.

### Histopathological evaluation

All histology slides were scanned using the Nano Zoomer Digital Pathology software, and image acquisition was performed using the NDP.view 2.4.26 software (NDP.view 2.4.26, Hamamatsu Photonics, Hamamatsu City, Japan). Villus height and crypt depth were measured in 10 well-oriented, villus-crypt units per intestinal section. Crypt depth was measured from the base of the crypt to the villus-crypt junction. Villus height was measured from the villus-crypt junction to the villus tip. For histological scoring, we used a system previously described (27, 28). Histological scoring was performed across all three intestinal segments, and data from each mouse were summed to obtain a total score. Histology was scored by an independent pathologist, blinded to treatment arm and genotype.

### Immunohistochemistry

A standard protocol was used for immunohistochemical staining of jejunal tissue. The primary antibodies were anti-mouse CD45 (30-F11) antigen (1:25; BD Pharmingen™, BD Biosciences, San Jose, CA, USA), cleaved Caspase-3 (Asp175) (1:50; Cell Signaling Technology, Danvers, MA, USA), and Ki-67 (SP 6) (1:50; Thermo Fisher Scientific, Waltham, MA USA). For CD45, a modified version of the PowerVision Detection System (polymer only) was used (Leica Biosystems, Newcastle, UK). For Casp-3 and Ki-67, the EnVision+ System-HRP (DAKO, Glostrup, Denmark) was used according to the manufacturer’s instructions.

Quantification of Caspase-3 positive cells was performed by counting the total number of positive cells in the crypts of each jejunal cross-section.

Quantification of CD45-positive cells was performed using ImageJ software (29), with quantification as the number of positive pixels (i.e., stained area) across the mucosal surface of the jejunum, including both villi and crypts.

Quantification of Ki-67-positive cells was performed in the crypts of the jejunum, since proliferation of new (epithelial) cells is expected to take place only in the crypts. Quantification was performed using ImageJ software (29), with quantification as the number of positive pixels as the metric.

### Serum citrulline

Serum concentrations of the non-essential amino acid citrulline were measured to evaluate enterocyte mass as a marker of small intestinal toxicity. A blood sample was collected by cardiac puncture. Samples were spun twice at 3,000 x g for 10 minutes and supernatants collected and stored at -80 °C. Concentration of citrulline was quantified on a UPLC-TQD MS (Waters) as previously described (30).

### Statistical analysis

Data were tested for normality using *qnorm* plots. Statistical significance of weight curves was evaluated by mixed effects analysis of paired data with Šidák’s multiple comparisons test between WT-Dox and KO-Dox groups for comparison of relative body weight changes from baseline on day 0 to day 7. Baseline (day 0) body weights were compared between genotypes using Welch’s unpaired t-test. For comparison of morphological changes in the intestine, citrulline levels, immunohistochemical assessments, and RT-qPCR data, statistical significance was determined by two-way ANOVA with uncorrected Fisher’s Least Significant Difference test. RT-qPCR data on *Sftpd*-expression were analyzed by t-test. Intestinal length measures were adjusted for baseline weight and expressed in cm/g. Villus/crypt ratio was calculated as a mean of three measurements obtained from the proximal, middle, and distal small intestine, respectively. A p-value < 0.05 was considered significant. Statistical analyses were performed, and graphs were made using GraphPad Prism 10 for MacOS (GraphPad Software, La Jolla, CA, USA).

### Ethics statement

The studies were carried out in accordance with the recommendations in the Guide for the Care and Use of Laboratory Animals of the National Institutes of Health. All animal procedures were approved by the Danish National Committee on Animal Experimentation (no. 2010/561-1760). The manuscript was prepared in accordance with the ARRIVE guidelines for reporting animal research (31).

## Results

### SP-D-deficient mice have significantly reduced body weight after doxorubicin challenge

At baseline (day 0), KO mice were significantly heavier than WT mice (KO 22.1 ± 1.2 g vs. WT 21.0 ± 1.4 g; mean difference 1.0 g [5%]; p = 0.002, Welch’s t-test).

**Figure 1:**
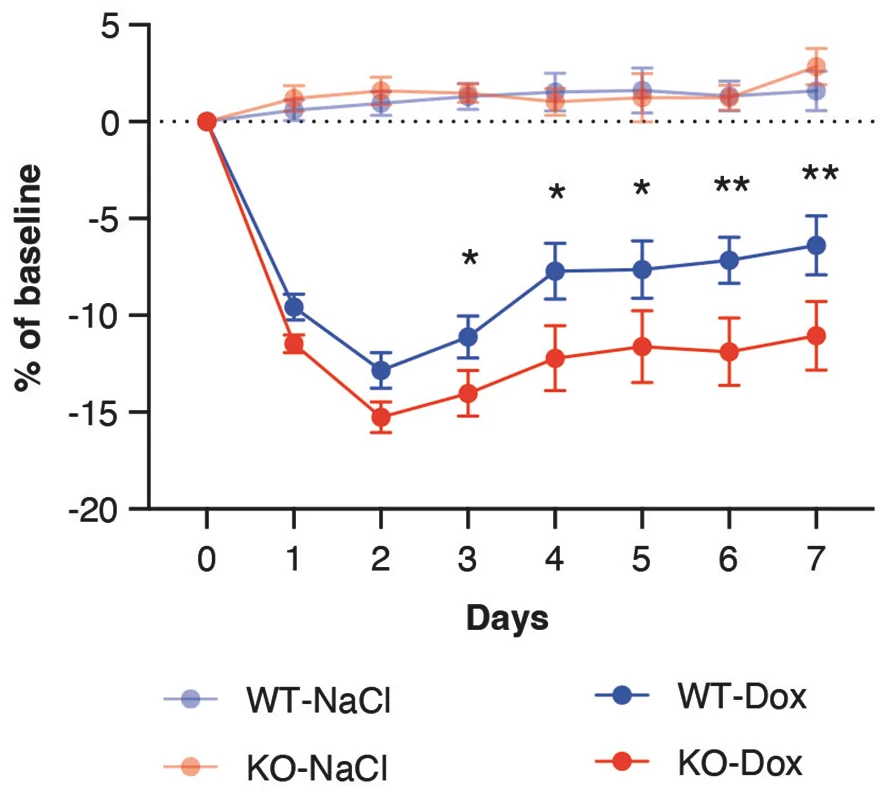
Relative body weight changes following doxorubicin-challenge. All animals from both the 3-day and 7-day experiments (total n = 66) are pooled and included in the relative weight development curves for the respective groups. WT = wild-type and KO = SP-D-deficient animals. Data points are shown as mean ± SEM. *p<0.05, **p<0.01, as estimated using mixed effects analysis of paired data with Šidák’s multiple comparisons test between WT-Dox (n=10(day3) +12(day7)) and KO-Dox (n=10(day3) +12(day7)) groups for comparison of relative body weight changes from baseline on day 0 to end study following saline (NaCl)- or doxorubicin (Dox)-challenge.

Doxorubicin challenge induced a significant body weight loss relative to saline treatment throughout the study period in both WT (p<0.0001) and KO animals (p<0.0001). This weight loss peaked above 10% by day 2 following challenge and did not return to baseline levels during the study. Doxorubicin-treated KO animals lost significantly more weight than doxorubicin-treated WT animals (p <0.05 – p <0.01) and remained with a weight loss above 10% until the end of the study (**Figure 1**).

### Doxorubicin-challenge induces mild morphological changes in the mouse intestine independently of SP-D

**Figure 2:**
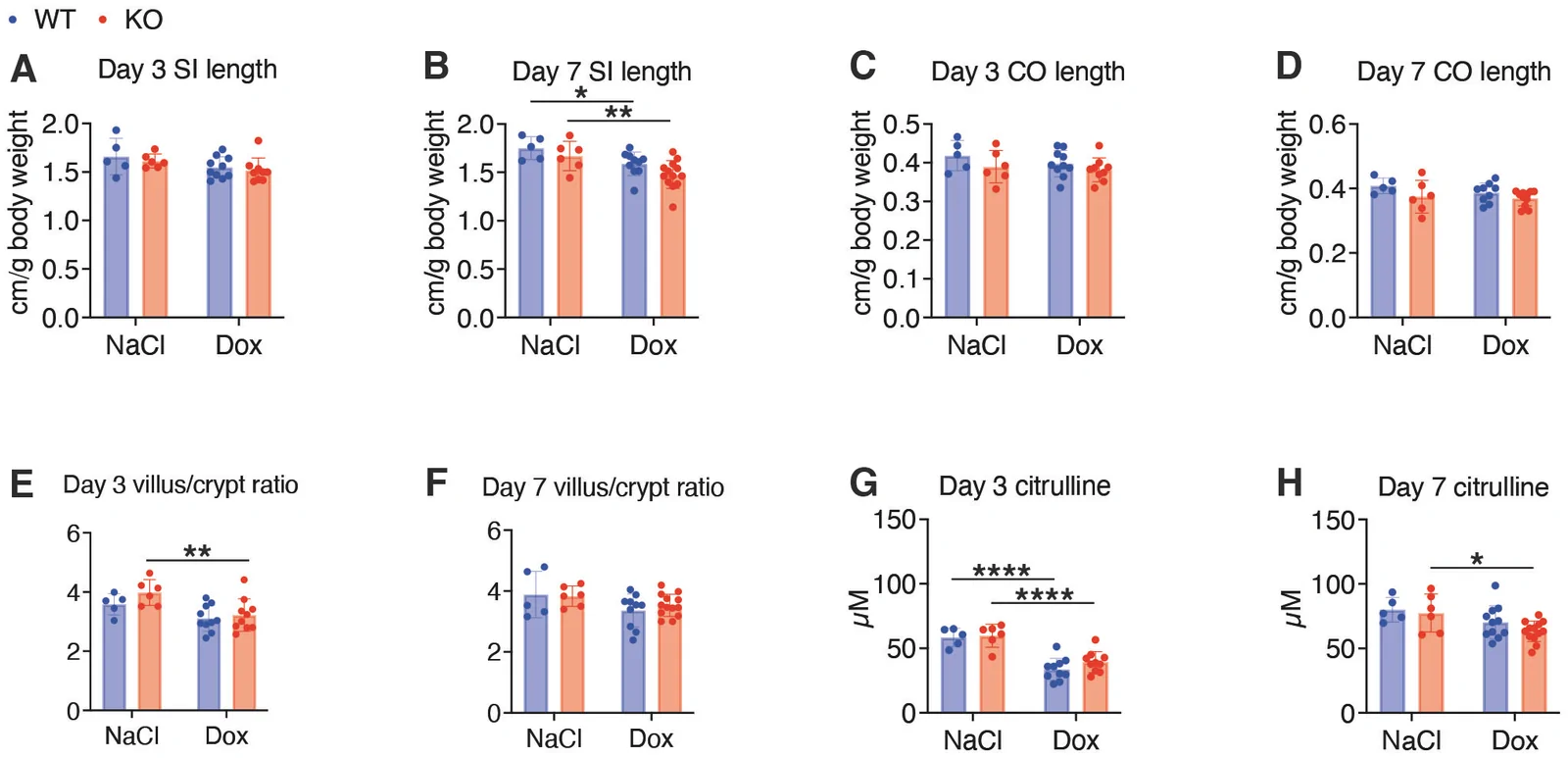
Intestinal morphological changes and plasma citrulline changes following doxorubicin-challenge. Measurements were obtained on days 3 and 7 following the doxorubicin challenge. WT = wild-type and KO = SP-D-deficient animals. Small intestinal (SI) length **(A)** day 3 and **(B)** day 7, colon (CO) length **(C)** day 3 and **(D)** day 7, villus-to-crypt ratio **(E)** day 3 and **(F)** day 7, and plasma citrulline levels **(G)** day 3 and **(H)** day 7. Data are presented as individual data points with mean ± SD. Statistical significance was determined by two-way ANOVA with uncorrected Fisher’s Least Significant Difference test, with *p < 0.05, **p < 0.01, and ****p < 0.0001.

Doxorubicin-challenged animals had significant shortening of the small intestine on day 7 as compared to saline controls (**Figure 2B**). No significant differences were observed for colon length (**Figures 2C, D**). The observed villus height/crypt depth ratios on day 3 were tendentially reduced by doxorubin-challenge in WT (*p* = 0.08) and significantly reduced in KO (**Figure 2E**). This change appeared to be transient, and no changes were observed in this ratio by day 7 (**Figure 2F**). As a marker of functional enterocyte mass, measurements of serum citrulline concentration revealed lower levels in the doxorubicin-treated animals on day 3 and day 7 (**Figures 2G, H**). No differences in these measures were observed among genotypes (**Figures 2A–H**). As anticipated, the histology score was affected in doxorubicin-treated animals, showing mean scores of median 1 (IQR 0-3) for KO and median 0.5 (IQR 0-5) for WT, respectively, in mice sacrificed on day 3. Total resolution was observed in mice sacrificed at day 7. No genotype-related differences were observed.

### Doxorubicin-challenge induces apoptosis and transient reduction of inflammatory cells in the mouse jejunum independent of SP-D

**Figure 3:**
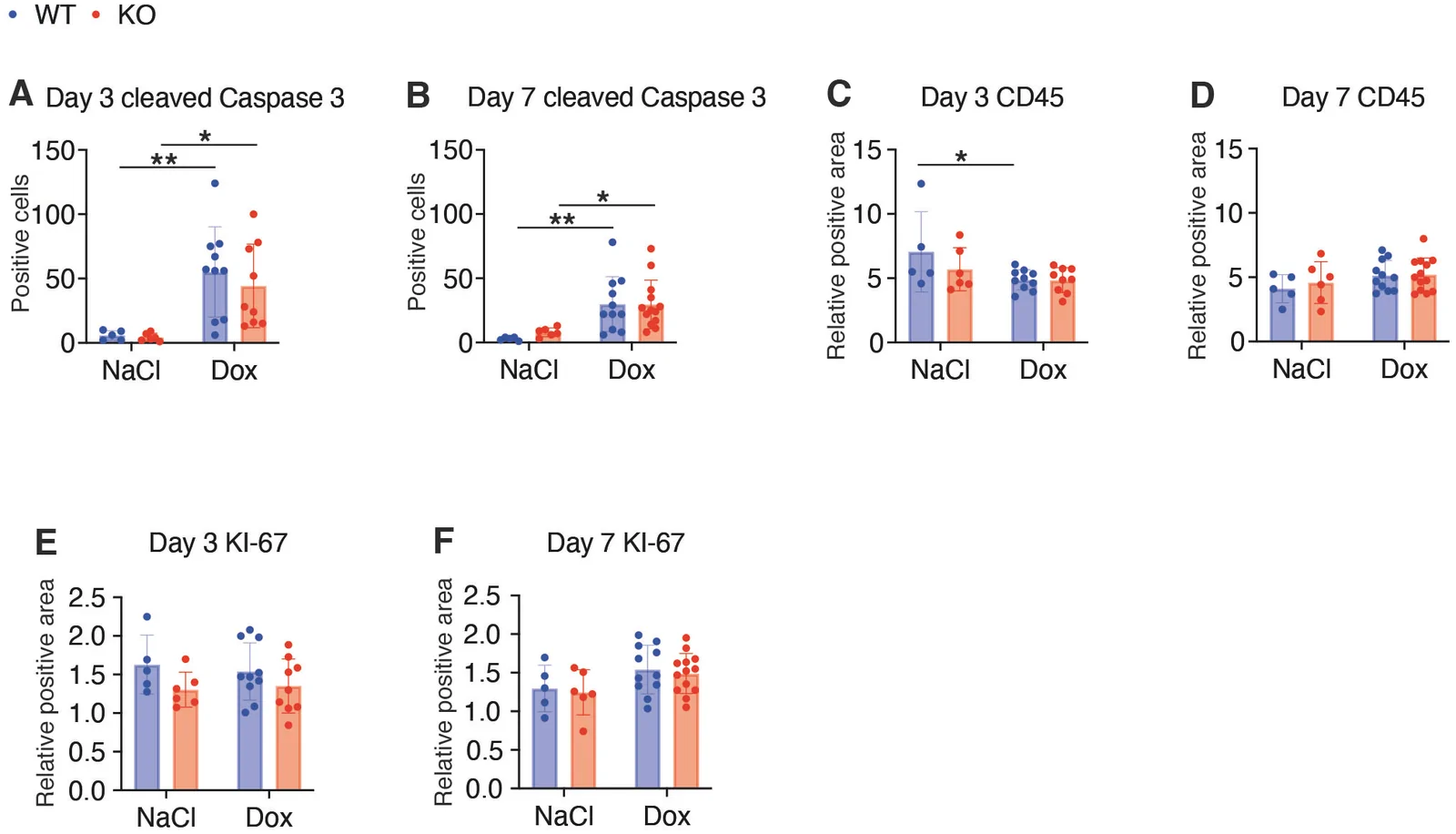
Apoptosis-, inflammation-, and proliferation-markers in the mouse jejunum following doxorubicin-challenge. Quantification of immunohistochemical markers was obtained on day 3 and day 7 following NaCl- or doxorubicin-challenge. WT = wild-type and KO = SP-D deficient animals. Apoptosis-marker cleaved Caspase-3 **(A)** day 3 and **(B)** day 7, Inflammatory cell marker CD45 **(C)** day 3 and **(D)** day 7, and cell proliferation marker KI-67 **(E)** day 3 and **(F)** day 7. Data are presented as individual data points with mean ± SD. Statistical significance was determined by two-way ANOVA with uncorrected Fisher’s Least Significant Difference test, with *p < 0.05, **p < 0.01.

Quantification of cleaved caspase-3 positive cells in the crypts revealed significantly increased numbers of apoptotic cells in the jejunum of ​doxorubicin-challenged mice at day 3 and day 7 as compared to saline controls (**Figures 3A, B**). Moreover, doxorubicin-challenged animals (WT) showed a transient and slightly decreased level of the CD45-marker of inflammatory cells as compared to saline-treated littermates (**Figures 3C, D**). No differences were observed among genotypes in these changes (**Figures 3A–D**). There was neither observed effects of doxorubicin nor genotype on the detection of the proliferative marker KI-67 (**Figures 3E, F**).

### RT-qPCR-based analyses show doxorubicin-induced changes in gene-expression related with inflammation, tissue injury/repair and apoptosis and effects of SP-D on gasdermin regulation

**Figure 4:**
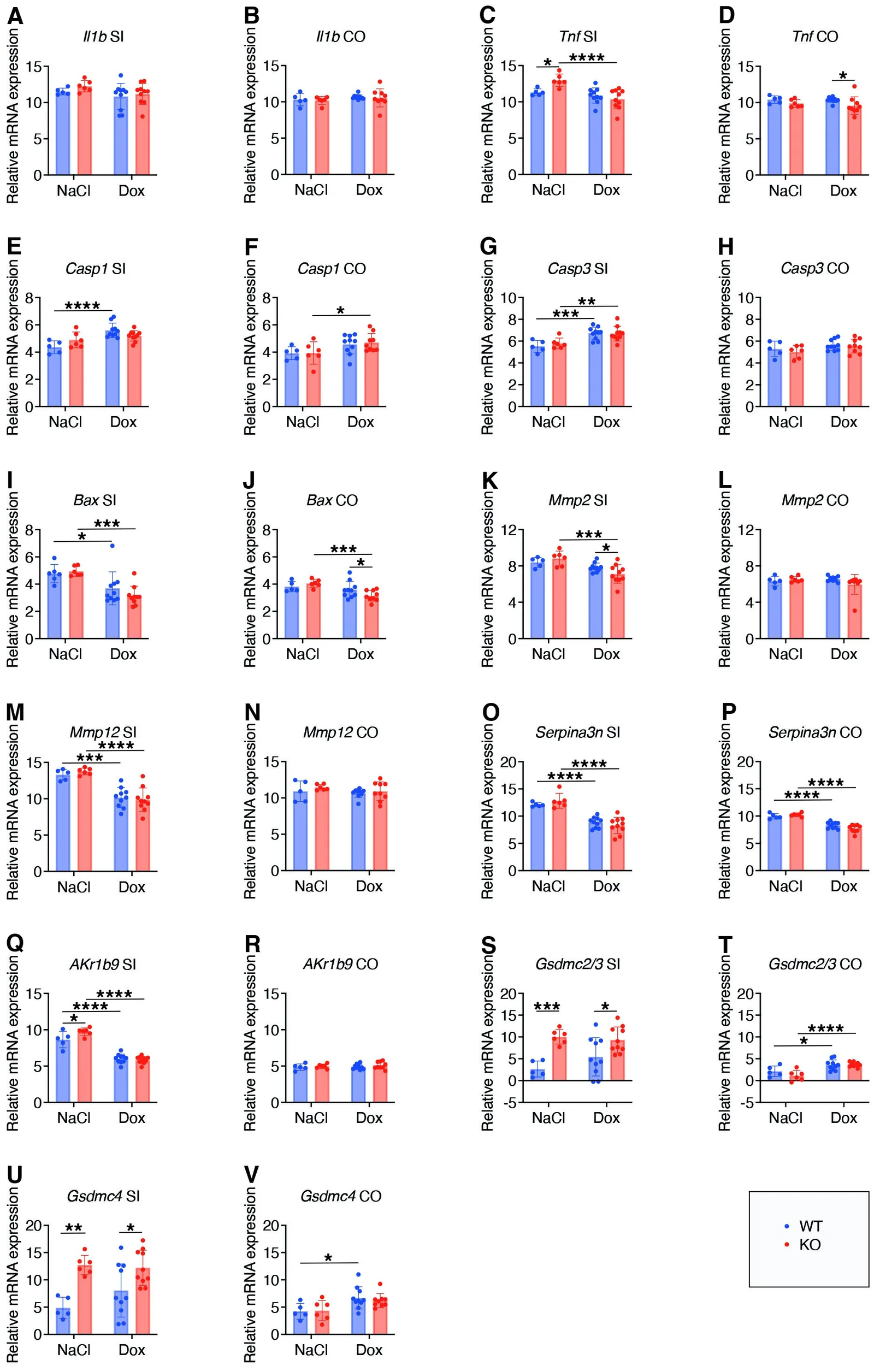
mRNA expression of selected genes in NaCl- or doxorubicin-challenged animals. Expressions of selected genes relative to *Gapdh* in the jejunum and colon in saline and doxorubicin- challenged mice as determined by RT-qPCR analysis. WT = wild-type and KO = SP-D deficient animals. SI = small intestine (jejunum) and CO = colon. Selected genes included **(A, B)** *Il1b,* **(C, D)** *Tnf*, **(E, F)** *Casp1*, **(G, H)** *Casp3*, **(I, J)** Bax, **(K, L)** *Mmp2*, **(M, N)** *Mmp12*, **(O, P)** *Serpina3n*, **(Q, R)** *AKr1b9*, **(S, T)** *Gsdmc2/3*, **(U, V)** *Gsdmc4* in jejunum and colon, respectively. Data in A-V are analyzed by two-way ANOVA with uncorrected Fisher’s Least Significant Difference test. Data are presented as individual data points with mean ± SD. *p<0.05, **p<0.01, ***p<0.001, ****p<0.0001.

*ll1b*-expression was not affected when assessed by RT-qPCR (**Figures 4A, B**). According to the RT-qPCR analysis, the most prominent changes after doxorubicin challenge included induction of *Casp1*, *Casp3* and *Gsdmc2/3* (**Figures 4E–G, T**) and downregulation of *Mmp2, Mmp12* and *AKr1b9* in the jejunum (**Figures 4K, M, Q**), while *Bax* and *Serpina3n* were downregulated both in jejunum and colon (**Figures 4I, J, O, P**). No corresponding regulation was observed in the colon for *Casp3, Mmp2, Mmp12* or *AKr1b9* (**Figures 4H, L, N, R**).

Minor changes in SP-D-dependent gene expression were observed for *Tnf* (**Figures 4C, D**), *Bax* (**Figure 4J**), *Mmp2* (**Figure 4K**), and *AKr1b9* (**Figure 4Q**). However, the most prominent effects of SP-D-ablation were increased jejunal expression of gasdermins (*Gsdmc2/3* and *Gsdmc4*) (**Figures 4S, U**).

### RT-qPCR-based analyses show no doxorubicin-induced changes in *Sftpd-*expression and detectable *Sftpd-*expression in both jejunum and colon

**Figure 5:**
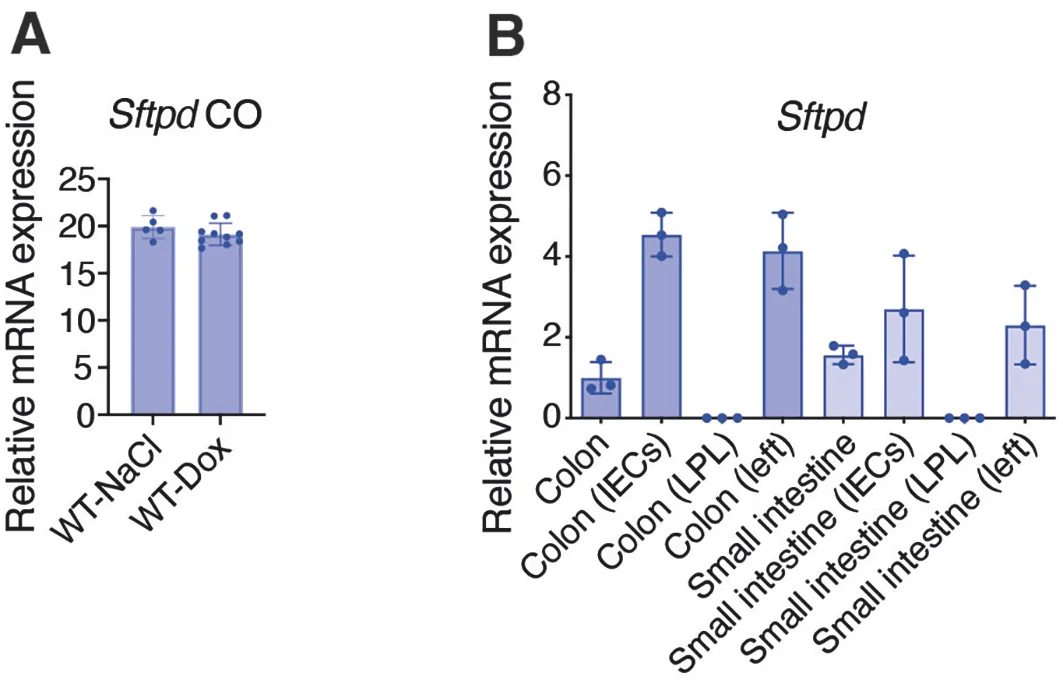
Relative mRNA expression of *Sftpd* in colon following doxorubicin challenge and across intestinal tissues and cell populations in unchallenged mice. Panels A and B originate from two independent experiments: The doxorubicin challenge cohort **(A)** and a separate set of unchallenged mice used for tissue and celltype profiling **(B)** — which accounts for the different reference genes used for normalization. **(A)** *Sftpd* expression relative to *Gapdh* in colon (CO) in saline and doxorubicin-challenged wild-type (WT) mice as determined by RT-qPCR analysis. **(B)** Relative *Sftpd* expression relative to *Gapdh* and *Tbp* in colon and small intestine, intestinal epithelial cells (IECs), lamina propria lymphocytes (LPL), and in tissue left after cellular isolation (left) in unchallenged mice. Data are presented as individual data points with mean ± SD.

We used colon samples to test if doxorubicin-challenge affected SP-D (*Sftpd*)-expression and found low expression (CT-values > 30) and no indication of doxorubicin-mediated regulation at this site (**Figure 5A**).

Finally, we tested *Sftpd-*expression in healthy mouse colon, jejunum, intestinal epithelial cells and lamina propria lymphocytes (**Figure 5B**). *Sftpd*-expression was detected in both colon and jejunum, and in the corresponding intestinal epithelial cells. As expected, there was no expression in lamina propria lymphocytes, while the tissue that was left after cellular isolation, and which was primarily comprising connective tissue, enteric neurons and smooth muscle from the intestine, also showed detectable *Sftpd*-expression.

## Discussion

Increasing evidence indicates that mucosal innate immune factors are important in the surveillance, protection, and homeostasis of mucosal surfaces during chemotherapy (32). They may contribute to crosstalk between the gut mucosal immune system and the general immunological response induced by chemotherapy-related gastrointestinal toxicity. Here, we studied the effect of SP-D, with suggested effects in gastrointestinal toxicity and inflammatory responses after chemotherapy (15, 33).

Among the toxic complications were hallmarks of chemotherapy-induced gastrointestinal toxicity, including decreased plasma citrulline levels, reductions in the length of the small intestine, and induced apoptosis. These doxorubicin-induced changes in the gastrointestinal tract were not affected by the ablation of SP-D. Thus, our findings of comparable gastrointestinal outcomes in SP-D KO and WT mice at both 3 and 7 days after doxorubicin administration suggest that SP-D deficiency has a limited effect on gastrointestinal toxicity after doxorubicin treatment, despite increased wasting.

Doxorubicin has previously been reported to induce both cachexia and sarcopenia in mice (34), effects that explain the induced wasting. SP-D deficiency has previously been associated with increased food intake and weight gain when fed ad libitum. These prior data suggested SP-D as a regulator of energy intake and body composition (35). Consistent with this, KO mice were heavier than WT mice already at baseline in the present study.

It has previously been demonstrated that SP-D-deficient mice exhibit intestinal dysbiosis and are therefore susceptible to dextran sulfate sodium-induced colitis (10). Such potential dysbiosis might affect feeding behavior, however food intake was not monitored in the present study, and the relationship between SP-D and body weight remains unknown and requires further mechanistic investigations.

Characteristic changes in chemotherapy-induced toxicity include shortening of villi and an increased villus/crypt ratio, indicating loss of absorptive epithelial cells (27). However, in this study, we observed a mild, transient loss of the villus-to-crypt ratio. Plasma citrulline, a marker of intestinal mass and function, was decreased on day 3 after doxorubicin administration, with partial regeneration by day 7. The partial regeneration was supported by the observed reduction in small intestinal length yet was not reflected by changes in the proliferative marker Ki-67. The lack of consistent SP-D-related effects on these parameters suggests that SP-D has limited effects on the morphological structure or regeneration of gastrointestinal epithelia following doxorubicin treatment. Although these intestinal parameters showed partial recovery by day 7, body weight remained reduced, consistent with systemic doxorubicin-induced cachexia and sarcopenia (34), which recover more slowly than the intestinal epithelium. The additional weight loss in SP-D KO mice indicates a further SP-D dependent contribution to the wasting phenotype.

Doxorubicin-induced regulation of several selected genes related to chemotherapy-induced mucositis, inflammation, apoptosis, and repair of damaged epithelium. Doxorubicin-treated KO mice did show slightly altered *Tnf, Bax*, and *Mmp2* mRNA expression after doxorubicin treatment, as evaluated by RT-qPCR. However, the most prominent finding was the upregulation of gasdermins in the small intestine of SP-D KO mice compared with WT mice. Members of the gasdermin protein family are modulators of pyroptosis, a specialized form of programmed cell death through pore-forming capacity in the plasma or mitochondrial membrane (36). Gasdermins have previously been implicated in doxorubicin-induced cell death in hematopoietic malignancies and in cardiotoxicity (37, 38). One genotype-associated finding was the increased expression of *Gsdmc2*, *Gsdmc3*, and *Gsdmc4* in SP-D-deficient mice. Gasdermins comprise a family of pore-forming proteins that can participate in inflammatory cell death pathways, including pyroptosis, and have been implicated in chemotherapy-induced cell death in other experimental settings (36–38). The increased expression of gasdermin genes therefore raises the possibility that SP-D deficiency influences pathways associated with regulated cell death in the intestinal mucosa. However, we did not observe corresponding increases in *Il1b* or *Casp1*, and we did not assess gasdermin protein expression or cleavage, inflammasome activation, or other direct markers of pyroptosis. Thus, the present findings do not establish that pyroptosis is functionally increased in SP-D-deficient mice. Rather, the data suggest that SP-D deficiency may alter the transcriptional regulation of gasdermin-associated pathways, potentially through mechanisms that are independent of canonical caspase-1-dependent inflammasome activation. Further studies assessing gasdermin protein activation and cleavage, inflammasome assembly and activity, and functional markers of pyroptosis are required to determine the biological significance of these transcriptional changes.

We confirmed SP-D mRNA expression in the intestinal tissues and intestinal epithelial cells of WT animals. Also, the remaining intestinal tissue after isolation of intestinal epithelial cells and lamina propria leukocytes showed *Sftpd*-expression. This residual tissue will include endothelial cells, which are known to express SP-D (39). However, we did not observe upregulation of SP-D after doxorubicin treatment, as seen in an earlier study of doxorubicin chemotherapy in piglets (15). This may indicate that the mouse is not ideal for studying doxorubicine-mediated effects on SP-D in the intestine. Limitations also included that we observed relatively modest pathological changes compared to earlier reports of doxorubicin-induced gastrointestinal toxicity in mice (27). A further limitation is that our analyses were restricted to the mRNA level. Quantification of SP-D at the protein level would be informative, but no validated method is currently available for quantifying murine SP-D; neither monoclonal antibodies developed in our laboratory nor commercially available polyclonal antibodies proved suitable for ELISA or semi-quantitative immunohistochemistry.

A limitation of the study was that only healthy mice were investigated. The current study found that SP-D deficiency had relatively little effect on the structural and molecular intestinal response to doxorubicin, but it was associated with greater body weight loss. In a tumor-bearing host, where nutritional status and systemic metabolism are already compromised, this effect could become more pronounced.

Moreover, we investigated the tissue responses in the early regenerative state. The different factors associated with mucositis are highly time-dependent, as also illustrated by this study, and thus our findings do not rule out the involvement of SP-D at an earlier (or later) time point.

## Conclusion

In this study, genetic deficiency of SP-D did not substantially alter the morphological, functional, inflammatory, or apoptotic manifestations of intestinal toxicity induced by a single dose of doxorubicin in healthy mice. Doxorubicin caused weight loss, reduced serum citrulline concentrations, shortening of the small intestine, increased epithelial apoptosis, and transient mucosal alterations, but these responses were largely independent of SP-D expression. Thus, under the experimental conditions examined, endogenous SP-D does not appear to provide major protection against the structural and functional intestinal toxicity associated with acute doxorubicin exposure.

Nevertheless, SP-D-deficient mice experienced significantly greater body weight loss, suggesting that SP-D may contribute to systemic responses to chemotherapy through mechanisms that extend beyond direct protection of the intestinal epithelium. Furthermore, the increased expression of *Gsdmc2, Gsdmc3*, and *Gsdmc4* in SP-D-deficient mice raises the possibility that SP-D modulates specific cell death-associated pathways in the intestine, although functional evidence is required to establish whether these changes reflect altered pyroptosis.

The detection of *Sftpd* expression in intestinal epithelial cells confirms that SP-D is locally produced in the mouse intestine. However, unlike our previous observations in doxorubicin-treated piglets, we found no evidence for induction of intestinal *Sftpd* expression following doxorubicin treatment in mice. This species-dependent difference, together with the relatively mild intestinal pathology observed in the present model, suggests that the contribution of SP-D to chemotherapy-induced mucositis may depend on species, disease severity, treatment regimen, and the timing of assessment.

Future studies should investigate SP-D protein expression and localization, intestinal barrier function, microbiota composition, and gasdermin activation across a broader time course. Importantly, studies in tumor-bearing animals are warranted to determine whether the role of SP-D differs in the clinically relevant setting of cancer-associated inflammation and chemotherapy. Such approaches may clarify whether SP-D represents a potential modifier of chemotherapy-induced gastrointestinal toxicity or whether its primary relevance lies in systemic metabolic and inflammatory responses to cancer treatment.

## Data Availability

The datasets presented in this study can be found in online repositories. The names of the repository/repositories and accession number(s) can be found below: https://www.ncbi.nlm.nih.gov/geo/query/acc.cgi?acc=GSE335354.
