# Supplementary material for: Limited effect of surfactant protein D ablation on doxorubicin-induced intestinal toxicity in mice
Source: Front Immunol. 2026 Sep 10;17:1913868. doi: 10.3389/fimmu.2026.1913868 (PMC13602119; doi:10.3389/fimmu.2026.1913868)
Supplement: Supplementary file 1 [file Table2.docx]

**Supplementary Table 2.**

| **Gene Symbol** | **Gene name** | **Entrez Gene ID** | **Assay ID** |
| --- | --- | --- | --- |
| *Gapdh* | Glyceraldehyde-3-phosphate dehydrogenase (house-keeping) | 14433 | Mm99999915_g1 |
| *Akr1b8* | Aldo-keto reductase family 1, member B8 | 14187 | Mm00484314_m1 |
| *Bax* | BCL2-associated X protein | 12028 | Mm00432051_m1 |
| *Casp1* | Caspase-1 | 12362 | Mm00438023_m1 |
| *Casp3* | Caspase-3 | 12367 | Mm01195085_m1 |
| *Gsdmc2/3* | Gasdermin C2/3 | 331063/270328 | Mm00626045_m1 |
| *Gsdmc4* | Gasdermin C4 | 74548 | Mm01194407_g1 |
| *Il1β* | Interleukin 1 beta | 16176 | Mm00434228_m1 |
| *Mmp12* | Matrix metallopeptidase 12 | 17381 | Mm00500554_m1 |
| *Mmp2* | Matrix metallopeptidase 2 | 17390 | Mm00439498_m1 |
| *Serpina3n* | Serine (or cysteine) peptidase inhibitor, clade A, member 3N | 20716 | Mm00776439_m1 |
| *Sftpd* | Surfactant associated protein D | 20390 | Mm00486060_m1 |
| *Tnf* | Tumor necrosis factor | 21926 | Mm00443258_m1 |

Gene symbol, assay ID and reference gene number of the target and housekeeping genes.
